# Lichen Planus Eruption Following Oxford-AstraZeneca COVID-19 Vaccine Administration: A Case Report and Review of Literature

**DOI:** 10.7759/cureus.22669

**Published:** 2022-02-27

**Authors:** Hamzeh M Alrawashdeh, Omar Al-Habahbeh, Abdallah Y Naser, Hashem Abu Serhan, Omar Hamdan, Kanar Sweiss, Yousef Aldalameh

**Affiliations:** 1 Department of Ophthalmology, Sharif Eye Centers, Irbid, JOR; 2 Department of Ophthalmology, Specialty Eye Hospital, Amman, JOR; 3 Department of Applied Pharmaceutical Sciences and Clinical Pharmacy, Faculty of Pharmacoepidemiology, Isra University, Amman, JOR; 4 Department of Ophthalmology, Islamic Hospital, Amman, JOR; 5 Department of Pathology and Laboratory Medicine, Mount Sinai Hospital, Toronto, CAN; 6 Department of Basic Pharmaceutical Sciences, Faculty of Pharmacy, Isra University, Amman, JOR

**Keywords:** sars-cov-2, oxford-astrazeneca vaccine, lichen planus, covid-19, coronavirus

## Abstract

Lichen planus is an autoimmune inflammatory disease that can be associated with infections, drugs, and vaccines. Recently, it has been reported to occur following mRNA-based COVID-19 vaccines, particularly the Pfizer/BioNTech vaccine. We present the first reported case of lichen planus that developed after five days following the administration of the first dose of the Oxford-AstraZeneca vaccine in a 46-year-old healthy male. The skin eruption was purple, ill-defined, non-scaly, itchy, and distributed over his face, abdomen, back, and legs. The clinical appearance of the skin eruption and histopathology confirmed the diagnosis of lichen planus. The skin lesions were not responding well to topical steroid and oral antihistamine treatment. Thus, the patient was commenced on systemic hydroxychloroquine.

The mechanism of lichen planus development following the administration of COVID-19 vaccines is unclear and needs more investigations and explanations. Healthcare providers should be aware of this possible adverse reaction following the administration of different severe acute respiratory syndrome coronavirus 2 (SARS-CoV-2) vaccines. The histopathological features of lichen planus in our case are different from those found in the lichenoid drug eruption. This finding indicates different pathophysiology that needs further investigation.

## Introduction

The first case of coronavirus disease 2019 (COVID-19) was reported in Wuhan, China, in December 2019. After that, a dramatic increase in reported cases was noticed in China and worldwide. The novel coronavirus, which was named later severe acute respiratory syndrome coronavirus 2 (SARS-CoV-2), was the implicated agent. The outbreak of SARS-CoV-2 prompted the World Health Organization (WHO) to declare COVID-19 a pandemic in March 2020 [1]. Along with the preventive methods to curb the spread of the disease globally, efforts were directed to develop safe and effective vaccines to control this emerging situation as soon as possible. In this context, different techniques were implemented to generate an effective vaccine for SARS-CoV-2. These included the classical inactivated vaccines (Sinovac Biotech, China), recombinant protein vaccines (Novavax, USA, and Sputnik V or Gam-COVID-Vac, Russa), viral vector-based vaccine/adenovirus-based vaccine (Oxford University/AstraZeneca, UK), nonreplicating viral vector vaccine (Johnson & Johnson/Janssen Biotech, the Netherlands-USA), and the mRNA-based vaccines (e.g., Pfizer/BioNTech, USA-Germany, and Moderna, USA) [2]. The efficacy of these vaccines was studied, and the results were encouraging. It was found that COVID-19 vaccines were highly effective in preventing SARS-CoV-2 infections [3]. Besides, infected patients who were previously vaccinated had a lower viral load and shorter duration of virus shedding than non-vaccinated patients [3,4].

Vaccines’ long-term adverse effects are still under monitoring, and it is a subject of interest nowadays. Recently, cutaneous manifestations such as diffuse urticaria, malar erythema, pityriasis rosea, herpes simplex flares, and lichen planus have been reported following Pfizer/BioNTech and Moderna (mRNA) COVID-19 vaccine administration [5-8]. No reactive dermatoses have been reported for other vaccines until April 2021, where, at that time, more than one billion doses of COVID-19 vaccine have been administered all over the world [8]. Lately, limited cases of reactive dermatoses following Oxford-AstraZeneca vaccine administration, such as erythema nodosum and pityriasis rosea-like eruption, were reported [9-11]. Nevertheless, there were no reported cases of lichen planus-like eruption.

Lichen planus is a chronic autoimmune-mediated inflammatory disease affecting T-cells. The definite pathogenesis of the disease is not well understood, but it is mostly related to the delayed hypersensitivity reaction of cytotoxic CD8+ lymphocytes [12]. It appears as intense pruritic red to violet flat-topped bumps on a regular skin surface, often localized to the extremities but may rarely be generalized [13]. Skin biopsy is the definitive diagnostic test for lichen planus [14].

Lichen planus or lichenoid-like eruption as a cutaneous manifestation following COVID-19 vaccines are rare, and the pathogenesis for its development is still unclear [15]. Lichen planus has been reported following Pfizer/BioNTech vaccine administration [7]. This report is the first that presents a case of a new-onset lichen planus triggered by the Oxford-AstraZeneca vaccine administration in a healthy young Caucasian male.

## Case presentation

**Figure 1:**
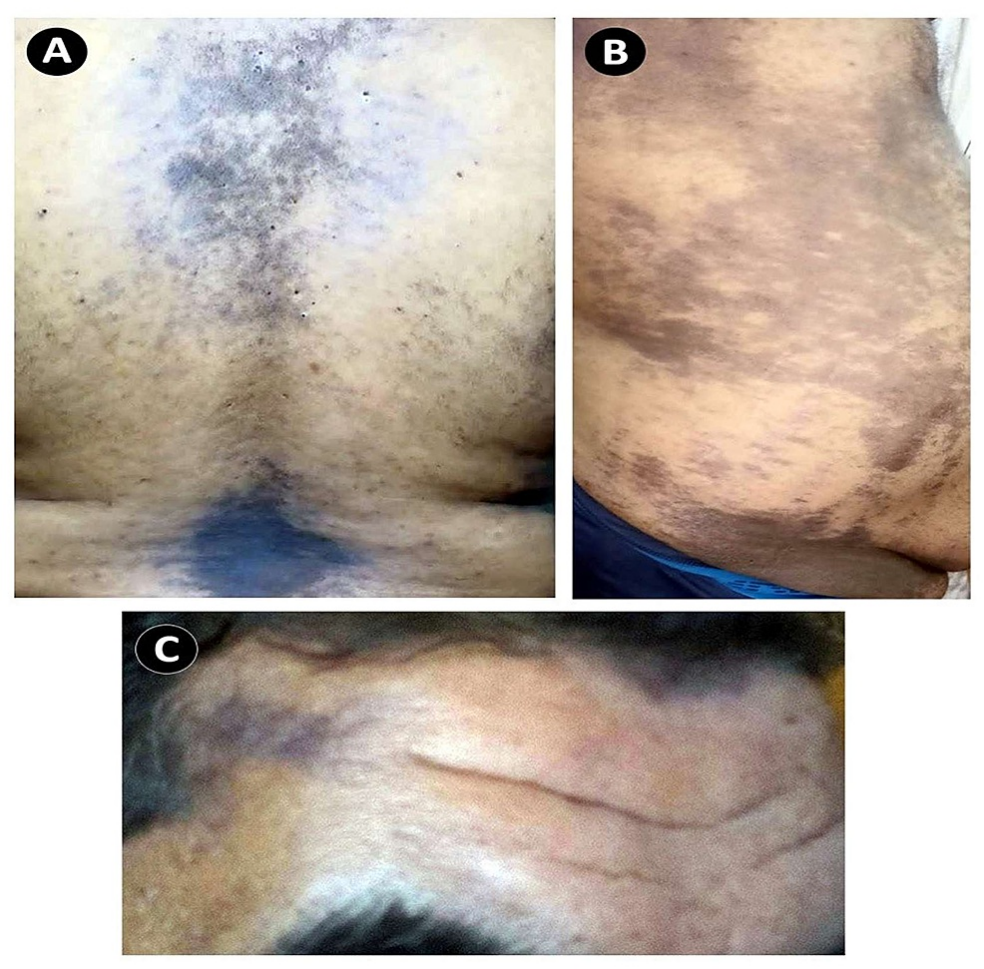
Gross appearance of lichen planus. Areas of purple, pruritic, and planar (flat) plaques on the back (A), abdomen (B), and face (C).

A 46-year-old male patient, without significant medical or surgical history, presented to our ophthalmology clinic for routine ophthalmic examination. Upon examination, the anterior and posterior segments and intraocular pressure were within normal limits in both eyes. Grossly pigmented skin lesions were noticed on the patient’s forehead. After meticulous history-taking, the patient stated that he is also suffering from itchy skin pigmented lesions over his abdomen, back, and legs for almost two months. He reported that his skin manifestations had appeared five days after the first dose of the Oxford-AstraZeneca vaccine. The skin eruption was purple, ill-defined, non-scaly, and distributed over his face (forehead), abdomen, back, and legs (Figure 1A-1C). The oral mucosa was not involved.

**Figure 2:**
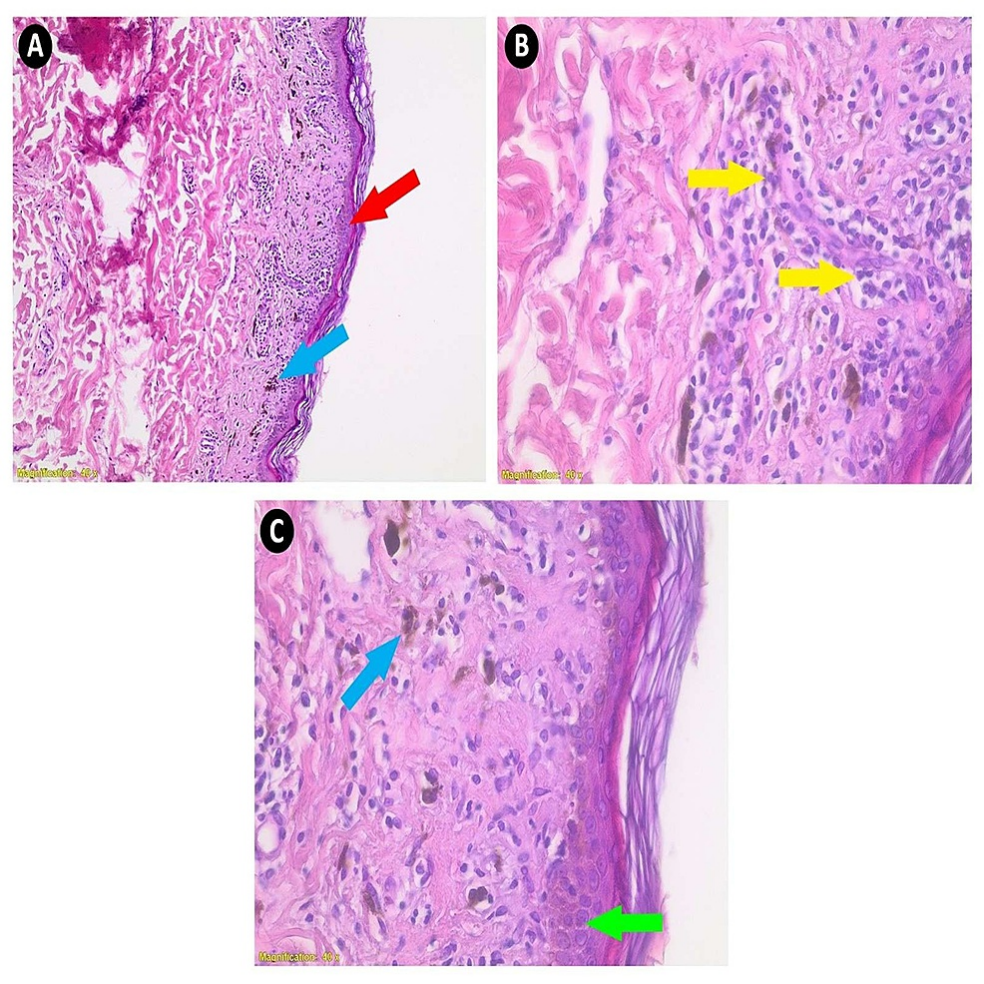
Histopathological features of the lesion. In low power (20×), there are minimal sawtooth-like rete ridges (red arrow) along with melanin pigment (brown deposition) within the macrophages of the upper layer of the dermis (blue arrow) without parakeratosis (A). In higher power (40×), a band-like chronic inflammatory cell infiltrates at the dermo-epidermal junction and perivascular space (yellow arrows). Most of those inflammatory cells are lymphocytes, which is one of the typical morphologic manifestations of lichen planus (B). Civatte body (green arrow) and melanin deposition (blue arrow) (C).

We referred the patient to a dermatologist for further evaluation and examination. All investigations of the patient, especially the hepatitis B and hepatitis C tests and antinuclear antibodies, were unremarkable. A 5-mm punch skin biopsy of the lesion was performed and sent to histopathology. The histopathological findings are shown in Figure 2A-2C.

The diagnosis of lichen planus was confirmed based on the clinical presentation and histopathological examination. The patient was treated with topical clobetasol propionate (0.1%) cream twice a day and oral antihistamines (hydroxyzine hydrochloride) 25 mg three times a day. Over four weeks, the condition showed mild improvement. The patient refused to take oral prednisone. Thus, he was started on hydroxychloroquine 200 mg twice daily and followed up regularly. The patient confirmed a significant reduction in pruritus after the second month of using hydroxychloroquine. However, the skin eruption showed minimal improvement.

## Discussion

Lichen planus is an autoimmune-mediated inflammatory disease, mostly related to T-cells, specifically cytotoxic (TH1) CD8+ cells that initiate an immune attack against basal keratinocytes. This mechanism is assisted by CD4+ helper T (TH2) cells via the secretion of TH1 cytokines, leading to apoptosis of basal keratinocytes [12,13,16]. Lichen planus is classified into cutaneous and mucosal according to the affected area. The prevalence of cutaneous lichen planus is equal in both genders, with a slight female predilection for oral lichen planus. The mean age at diagnosis of lichen planus is between 50 and 60 years for oral type and between 40 and 45 years for the cutaneous type. The disease is rare in children [17].

Lichen planus appears as an intense pruritic red to violet flat-topped bumps on a regular skin surface. It is often described using the “six P’s” (purple, pruritic, polygonal, planar, papules, and plaques) and characterized by the classic Wickham striae (fine white or gray lines or dots seen on the top of the papular rash and oral mucosal lesions). The distribution of the rash is often localized to the extremities but may rarely be generalized. In addition to the skin, nails can also be affected; nail thinning, scarring, and even complete loss of the nail can be seen. It can also affect the vagina, penis, and hair follicles [13]. Lichen planus can be associated with several infections such as hepatitis B virus, hepatitis C virus, and varicella-zoster and medications such as angiotensin-converting enzyme inhibitors, antimalarials, beta-blockers, gold, lithium, carbamazepine, tetracyclines, and vaccines, especially hepatitis B virus vaccine [13,18,19]. Recently, the Pfizer/BioNTech vaccine was known to be associated with lichen planus eruption [18].

The classical morphology for lichen planus should reveal hyperkeratosis, acanthosis, wedge-shaped hypergranulosis, melanin deposition within the macrophages of the dermis (pigment incontinence), sawtooth-like appearance of the rete ridges, and severe band-like lymphohistiocytic infiltrate effacing the dermo-epidermal junction. Sometimes, Civatte or cytoid bodies, basal cell squamatization (flattening of cells), and artifactual cleft formation are present between the epidermis and papillary dermis (Max Joseph space). Parakeratosis is generally absent [20,21].

There are many entities for lichen planus based on the clinical-morphologic evidence [20,21]: atrophic lichen planus (no acanthosis, fewer Civatte bodies, more pigment incontinence, and minimal inflammation), which represents our case; hypertrophic lichen planus (marked hyperkeratosis and acanthosis (pseudoepitheliomatous hyperplasia)); ulcerative (erosive) lichen planus (epidermal ulceration with lichen planus changes at the ulcer margins); pigmentosus (lymphocytic infiltrate tends to be deeper, with dermal melanin incontinence); and actinic lichen planus (dermal melanin incontinence and focal parakeratosis with milder lymphocytic infiltrate).

The most common differential diagnosis for lichen planus is lichenoid drug eruption, in which there are lichenoid interface infiltrates with basal cell squamatization and Civatte bodies. Lichenoid drug reaction is associated with a high number of necrotic keratinocytes, plasma cells, and eosinophils within inflammatory cells. In addition, a correlation was found between the number of granzyme B+ cells and apoptotic keratinocytes and between the number of T-cells and CD8+ cells in lichenoid drug reactions, suggesting that these cells may have a major role in lichenoid drug reactions and somehow have distinct pathogenesis [22]. Distinguishing features from lichen planus are generally based on the clinical history of potential medication and the presence of eosinophils, and sometimes parakeratosis. However, these two histological features are not present in our case. The presence of eosinophils favors lichenoid drug reaction than conventional lichen planus (eosinophils can be seen in hypertrophic lichen planus, which is not seen in our case because there is no striking hyperkeratosis and hypergranulosis seen in this type of lichen planus) [23].

In the literature, there are only a few reported cases of new-onset lichen planus arising after the Pfizer/BioNTech COVID-19 vaccine (mRNA vaccine) administration, and the definite mechanism that stands behind it is not yet fully understood [7,16]. These reported cases indicated that lichen planus might arise following the first or second dose of the vaccine with scarce eosinophils in the dermis on histopathology [7,24,25]. In our case, the patient developed lichen planus five days following the first dose of the Oxford-AstraZeneca vaccine, and there were no eosinophils on histopathology.

The mRNA vaccine elicits an immune reaction after being injected into the human body and inserted into the cytoplasm of the host cells. The mRNA is translated, and the forming proteins achieve the cellular membrane in two complexes, the MHC-1 and MHC-2. The MHC-2 is responsible for the activation of T-helper cells and the production of cytokines, such as IL-2, IL-4, and IL-5, which aid B-cell differentiation into antibody-producing plasma cells. The MHC-1 interacts with T-cytotoxic cells and produces CD8+ proteins [26]. The Oxford-AstraZeneca vaccine is a modified DNA Chimpanzee adenovirus vector that does not generate an immune response to the adenovirus itself but only to the viral protein encoded in the host DNA. Following administration, it gets released in the cytoplasm of the host cells and then converted into mRNA, leading to the formation of MHC-1 and MHC-2 complexes similar to the mRNA vaccine [21]. It elicits a process of protein translation inside the cell, activating T-cells, B-cells, plasma cells, and antibodies, providing the host with the required immunity [26].

A proposed explanation for the mechanism of lichen planus following the Oxford-AstraZeneca vaccine is that the vaccination may have stimulated a type 1 T helper (TH1) cell response and a subsequent various cytokine secretion that could play a key role in the development of this condition [7]. Moreover, antibody-dependent enhancement (ADE) or vaccine-enhanced disease (VED) is considered a concern for SARS-CoV-2 vaccine development. High-affinity antibodies against epitopes of viral particles may result in the elimination of the virus. However, heterotypic antibodies or of suboptimal concentration will increase disease severity [27]. In the case of COVID-19 vaccines, ADE may stimulate cutaneous manifestations. Some studies demonstrate the presence of a correlation between ADE and increased disease severity post-vaccination [27,28]. These suggestions may explain lichen planus development following Oxford-AstraZeneca vaccine administration in our case.

The treatment of lichen planus includes topical steroids as initial treatment. Systemic steroids can also be used if topical treatment fails. In addition, phototherapy and many other medications, including retinoids, griseofulvin, metronidazole, efalizumab, thalidomide, sulfasalazine, mycophenolate mofetil, azathioprine, and hydroxychloroquine, can be used, but not as first-line treatment [17,21].

## Conclusions

Lichen planus can develop following some COVID-19 vaccines such as Oxford-AstraZeneca and Pfizer/BioNTech vaccines. The cutaneous eruption can arise following the first dose of the SARS-CoV-2 vaccines. The histopathological features of lichen planus, induced by the vaccine, are different from those found in the lichenoid drug eruption provoked by medications. This observation reveals that pathophysiology is different and needs further investigation and evaluation. In the era of SARS-CoV-2 vaccines, physicians should be aware of the possible adverse reactions associated with the vaccines, such as lichen planus and other cutaneous manifestations. Lichen planus induced by COVID-19 vaccines responds to the same regimens used for the traditional disease.
